# TSG Extends the Longevity of *Caenorhabditis elegans* by Targeting the DAF-16/SKN-1/SIR-2.1-Mediated Mitochondrial Quality Control Process

**DOI:** 10.3390/antiox13091086

**Published:** 2024-09-04

**Authors:** Menglu Sun, Congmin Wei, Yehui Gao, Xinyan Chen, Kaixin Zhong, Yingzi Li, Zhou Yang, Yihuai Gao, Hongbing Wang

**Affiliations:** 1 Institute for Regenerative Medicine, Shanghai East Hospital, School of Life Sciences and Technology, Tongji University, Shanghai 200092, China; 2 Tongji Alpha Natural Medicine Research Institute, Tongji University, Shanghai 200070, China

**Keywords:** 2,3,5,4′-Tetrahydroxystilbene-2-*O*-*β*-D-glucoside, *Caenorhabditis elegans*, mitochondrial function, aging, neurodegenerative diseases

## Abstract

The improvement of mitochondrial function is described as a strategy for alleviating oxidative stress and intervening in the aging process. 2,3,5,4′-Tetrahydroxystilbene-2-*O*-*β*-D-glucoside (TSG) is one of the major bioactive components isolated from *Polygonum multiflorum* Thunb, and it exhibits multiple activities, including antioxidant and anti-inflammatory effects. In this study, we found that 200 μM TSG significantly extended the mean lifespan of *Caenorhabditis elegans* by 16.48% and improved health status by delaying age-associated physiological decline in worms. The longevity prolongation effect of TSG depended on the regulation of the mitochondrial quality control process mediated by DAF-16/FOXO, SKN-1/Nrf2 and SIR-2.1/SIRT1 to improve mitochondrial function. Moreover, TSG treatment obviously alleviated the proteotoxicity of β-amyloid and tau proteins in worms. Our findings indicated that TSG is a promising natural product for preventing aging and treating aging-associated neurodegenerative diseases by regulating the mitochondrial quality control process to improve mitochondrial function.

## 1. Introduction

Aging is an irreversible process in which biological function gradually deteriorates with age [1]. Oxidative stress is widely considered to be one of the critical causes of aging, and contributes to mitochondrial dysfunction characterized by mitochondrial DNA damage, increasing electron leakage from the mitochondrial electron transfer chain (ETC) and decreasing mitochondrial membrane potential and ATP, which can be improved by mediating the mitochondrial quality control (MQC) process through the mitochondrial unfolded protein response (UPR^mt^), mitochondrial biogenesis and mitophagy [2,3]. Therefore, drug development targeting antioxidant defense and mitochondrial homeostasis is crucial for the treatment of aging and age-associated diseases.

2,3,5,4′-Tetrahydroxystilbene-2-*O*-*β*-D-glucoside (TSG), a polyhydroxy stilbene, is the main bioactive ingredient of *Polygonum multiflorum* Thunb. Previous studies have shown that TSG possesses multiple pharmacological properties, including antioxidative, anti-inflammatory, antiatherosclerotic and neuroprotective effects [4]. More recently, we demonstrated that a 60% ethanol fraction of *Polygonum multiflorum* Thunb (PMT-E) could significantly increase the mean lifespan and inhibit the toxicity induced by β-amyloid (Aβ) in transgenic *Caenorhabditis elegans* [5]. Furthermore, given that TSG was identified as the major compound in PMT-E according to HPLC analysis, we continued to study the effect and mechanism of action of TSG on longevity and neurodegenerative diseases, as reported in this article.

We found that TSG not only upregulated the expression of antioxidant enzymes and reduced ROS levels, but also improved mitochondrial function by enhancing mitophagy and mitochondrial biogenesis processes in a DAF-16/SKN-1/SIR-2.1-dependent manner, thus revealing the biological mechanisms of TSG in antioxidant stress, delaying the senescence of worms and preventing neurodegenerative diseases.

## 2. Materials and Methods

### 2.1. Strains

*C. elegans* strains were cultured at 20 °C on nematode growth media (NGM) seeded with *E. coli* OP50, with the exception of CL4176, CL2355 and CL2331, which were maintained at 16 °C. The strains used in this study were provided by the Caenorhabditis Genetics Center (CGC) and are listed in Supplementary Table S1. For the generation of hybrid progenies of the CL4176 and SJ4103 strains, the L4 stage SJ4103 nematodes were exposed to 30 °C for 5 h to produce males, which were subsequently mated with L4 hermaphrodites of the CL4176 strains. Homozygotes were screened for the presence of GFP in body wall muscle and a rotation phenotype.

### 2.2. Lifespan Assay

Age-synchronized L1 larvae were hatched overnight from eggs, which were isolated from gravid adults by hypochlorite bleaching. The L1 larvae were then transferred to 96-well plates containing S-complete culture medium and *E. coli* OP50. After 45–48 h, 5-fluoro-2′-deoxyuridine (Sigma, Darmstadt, Germany) was added at a final concentration of 180 μM to sterilize the animals, and different concentrations of TSG (100, 200, and 400 μM) dissolved in 0.3% DMSO were added when the L4 stage was reached. Worms were scored as dead or alive every two days until all the animals died.

### 2.3. Phenotype Assays

#### 2.3.1. Body Bending

For the mobility test, N2 worms were pretreated for 4 or 8 days as described for the lifespan analysis. At least twenty nematodes per condition were transferred to individual NGM plates without OP50, and the number of body bends within 30 s was counted.

#### 2.3.2. Pharyngeal Pumping

Synchronized N2 L4 larvae were cultured in liquid medium supplemented with or without 200 μM TSG. After 4 or 8 d of adulthood, pharyngeal pumping was quantified by recording the movement of the pharynx bulb at 15 s intervals. For each measurement, twenty worms were randomly examined among the population.

#### 2.3.3. Lipofuscin Assay

Synchronized N2 L4 larvae were cultured in liquid medium with or without 200 μM TSG. At day 4 of adulthood, worms were washed three times with M9 buffer and then transferred to new NGM plates without *E. coli* OP50. Approximately 20 animals were mounted on slides containing 4% paraformaldehyde to visualize intestinal fluorescence under a fluorescence microscope (blue excitation light, 405–488 nm). Lipofuscin levels were quantified using ImageJ software (version 1.53c) by determining the fluorescence intensity and subtracting the background [6].

### 2.4. Stress Resistance Assays

#### 2.4.1. Paraquat or Rotenone Stress Resistance

Synchronized L4 larvae were exposed to DMSO or 200 μM TSG until adult day 4, after which they were subsequently transferred to a new 96-well plate containing 50 mM paraquat or 50 μM rotenone [7]. The survival was scored daily or recorded after 24 h. At least 50 animals per condition were examined.

#### 2.4.2. Thermotolerance Assay

For the heat tolerance assay, thirty 4-day-old adult worms were pretreated with 200 μM TSG and their controls were placed on NGM plates at 35 °C for 4 h and then transferred to 20 °C again. The number of worms was monitored daily for death.

#### 2.4.3. High-Glucose Stress Assay

Synchronized L4 larvae were cultured with or without 200 μM TSG as described for the lifespan assay, and glucose was added to a final concentration of 50 mM [7]. On day 4 of adulthood, body bends and mitochondrial membrane potential were detected.

### 2.5. ROS Assay

Four-day-old adult worms were washed three times with M9 buffer to remove residual bacteria and transferred to black 96-well plates containing M9 buffer (10 worms/well). Then, the fluorescent probe H2DCF-DA was added to a final concentration of 50 μM. Samples were measured every 20 min for 2 h in a SpectraMax^®^ ID5 microplate reader (excitation wavelength of 485 nm; emission wavelength of 535 nm) at 37 °C.

### 2.6. Fluorescence Assay

Synchronized L4 larvae of transgenic strains expressing the reporter genes *sod-3p::GFP*, *gst-4p::GFP* or *sqst-1p::GFP* were cultured in the absence or presence of 200 μM TSG for 96 h, after which the fluorescence images were collected and measured as described previously [8].

### 2.7. DAF-16 and SKN-1 Nuclear Translocation Assays

Fluorescence photographs were obtained as described above, the subcellular localization of DAF-16 (TJ356) and SKN-1 (LD1) was classified into nuclear, cytosolic, and intermediary categories. Approximately 30 worms were measured in every group.

### 2.8. Quantitative RT-PCR Assay

Approximately 500 synchronized worms were raised until they developed into 4-day-old adults. Total RNA was extracted with TRIzol (Tiangen, Beijing, China) and reverse transcribed into cDNA using a cDNA Synthesis Supermix Kit (B24403, Bimake, Houston, TX, USA). qRT–PCR was performed using SYBR Green qPCR Master Mix (B21202, Bimake, Houston, TX, USA) on a LightCycler 96 Real-Time PCR System (Roche, Rotkreuz, Switzerland). The expression levels of the target genes were calculated by the 2^-∆∆Ct^ method and normalized to that of actin-1 (act-1). The primer sequences are listed in Supplementary Table S2.

### 2.9. Paralysis Assay

The synchronized L1-stage CL4176 strains (35–40 animals per condition) were cultured on NGM plates containing either DMSO or 200 μM TSG at 16 °C for 36 h and then upshifted to 23 °C to induce the expression of Aβ. After 27 h of temperature conversion, the number of paralyzed nematodes was recorded every hour until all worms were paralyzed. Worms were scored as paralyzed if they moved only their head in response to touching stimulation with a platinum wire [9].

### 2.10. Chemotaxis Assay

Synchronized L1 transgenic *C. elegans* CL2355 were treated with or without 200 μM TSG at 16 °C until they reached the L3 stage (approximately 36 h), followed by an additional 36 h at 23 °C [10]. Approximately 100 worms were collected and washed three times with M9 buffer, then placed near the center of a NGM plate without food. Then, 10 μL of 1 M sodium acetate along with sodium azide as an attractant were added approximately 0.5 cm from the edge of the agar plate. On the opposite spot, 10 μL of water and sodium azide were added. Assay plates were scored after 60 min and the specific chemotaxis index (CI) was calculated as follows: number of animals at attractant - number of animals at water location/total number of animals in assay.

### 2.11. Serotonin Sensitivity Assay

Synchronized transgenic *C. elegans* CL2355 were cultured as described in the chemotaxis assay and then washed with M9 buffer for three times. Approximately 30 worms were transferred into a 96-well plate containing 150 μL of the 5 mg/mL serotonin solution (10 per well). The number of active nematodes in each group was counted after 24 h.

### 2.12. Behavior Analysis of Transgenic C. elegans Expressing Tau Protein

#### 2.12.1. Locomotion Speed Assay

Approximately 50 synchronized BR5706 adults per condition were transferred to NGM plates without bacteria to acclimate for 1 h. The movement distance of *C. elegans* within 10 s was performed with WormLab software (version 2018.1.1, MBF Bioscience, Williston, ND, USA). Only worms that moved continuously and independently during acquisition were analyzed.

#### 2.12.2. Liquid Thrashing Assay

The thrashing assay is also known as a swimming test. In brief, a single 4-day-old VH254 adult was placed in 50 μL M9 buffer on a microscope slide. The number of body bends in 10 s was counted, and at least 30 worms were measured for each treatment.

### 2.13. Western Blotting

Synchronized 4-day-old adult animals were washed with PBS and then homogenized in ice-cold RIPA lysis buffer supplemented with phosphatase and protease inhibitors, followed by centrifugation for 10 min at 12,000 rpm to remove insoluble debris. The total protein content was measured by the BCA method, and the samples were boiled with loading buffer at 100 °C for 5 min. Forty micrograms of each sample was subjected to Tris-Tricine gel electrophoresis and transferred to a PVDF membrane. After blocking the membranes for 1 h with 5% milk, they were incubated overnight with anti-SIR-2.1 (1:3000; Thermo Fisher Scientific, Waltham, MA, USA, PA1-16933), and anti-GAPDH (1:5000; Proteintech, Rosemont, IL, USA, 60004-1-Ig) was used as an internal reference. The membranes were incubated with conjugating secondary antibodies. The blots were visualized by enhanced chemiluminescence.

### 2.14. RNA Interference (RNAi) Assay

*E. coli* HT115 bacteria expressing double-stranded RNA (dsRNA) were generated and fed to *C. elegans* to suppress the *sir-2.1* gene as previously described [11]. Briefly, synchronized L1 stage CL4176 worms were transferred to NGM plates containing 100 μg/mL ampicillin, 1 mM IPTG and *E. coli* HT115 with the empty vector L4440 or the *sir-2.1* gene. The RNA was extracted and detected by qPCR after culturing for 2–3 generations. A significant decrease in the expression of *sir-2.1* indicated that the target gene was successfully silenced and could be used in subsequent paralysis experiments.

### 2.15. Mitochondrial Membrane Potential (ΔΨ) Assay

For staining, synchronized 4-day-old adult animals were incubated in the presence of rhodamine 6G for 5 h. Twenty to thirty adults were transferred to a new NGM plate and allowed to acclimate to the plate for 5 min to remove the excess rhodamine 6G [12]. The stained worms were then formalized with 4% paraformaldehyde and visualized using a fluorescence microscope as previously described.

### 2.16. ATP Level Assay

The ATP level in the adult worms was assayed using an ATP Assay Kit (Beyotime, Shanghai, China). Five hundred 4-day-old adult animals were homogenized in ice-cold ATP lysis buffer, and a portion of the supernatant was used for protein determination after centrifugation at 12,000 rpm for 5 min at 4 °C. The luminescence signal was recorded using a SpectraMax M5 fluorescent plate reader (Molecular Devices, Sunnyvale, CA, USA), and ATP level was normalized to total protein content.

### 2.17. NAD^+^ Level Assay

NAD^+^ level was determined using an NAD^+^ quantification kit according to the manufacturer’s instructions. Pretreated 4-day-old worms were homogenized in acid lysis buffer and boiled for 5 min. Then, the extract was centrifuged at 10,000× *g* for 10 min at 4 °C, and the supernatant was transferred to another tube. An equal volume of alkaline solution was added for neutralization, and the supernatant was subjected to NAD^+^ detection according to the manufacturer’s instructions after repeated centrifugation. The data were normalized to the total protein concentration measured using a BCA assay.

### 2.18. Mitochondrial DNA (mtDNA) Copy Number Assay

The copy number of mtDNA was quantified using nuclear DNA (nDNA) as an internal reference. Single adult 4-day-old nematodes were transferred to a 25 μL reaction system containing ExTaq Buffer, 2.5 mM dNTP mixture, primers targeting mtDNA and nDNA, 200 μg/mL proteinase K and nuclease-free water. After inactivating Proteinase K at 95 °C for 10 min, then incubating at 60 °C for 20 min to lyse the worms, the ExTaq enzyme was added. The PCR amplification program was as follows: 94 °C for 5 min, followed by 36 cycles of 94 °C for 30 s, 60 °C for 45 s, and 72 °C for 50 s, and a final extension at 72 °C for 10 min. The obtained PCR products were quantified, and the copy number of mtDNA was normalized to that of the nDNA.

### 2.19. Statistical Analysis

GraphPad Prism 8.0 and ImageJ (version 1.53c) were used for the statistical analysis. For lifespan and paralysis trials, Kaplan–Meier survival curves were generated, and *p* values were calculated using the log-rank test. To compare two groups, the significance of the data was analyzed by Student’s test. Differences were considered significant at *p* < 0.05.

## 3. Results

### 3.1. TSG Extended the Lifespan and Improved the Health Status of C. elegans

**Figure 1:**
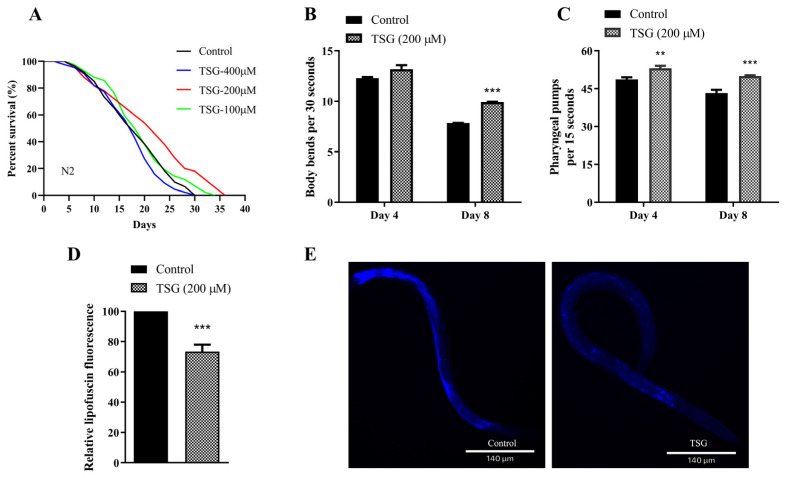
TSG extended the lifespan and improved the physiological indices of *C. elegans*. (**A**) TSG extended the lifespan of N2 wild-type *C. elegans* (control, mean 17.66 ± 0.37 days; 100 μM TSG, mean 19.43 ± 0.06; 200 μM TSG, mean 20.57 ± 0.41; 400 μM TSG, mean 18.15 ± 0.34). (**B**) TSG elevated the locomotor ability of worms. The number of body bends in 30 s was detected on the 4th and 8th days. (**C**) TSG increased the pharyngeal pumping frequency of worms. The pharyngeal pumping rates of individuals were measured for 15 s on the 4th and 8th days. (**D**,**E**) TSG inhibited lipofuscin content in 4-day-old nematodes. Representative images and fluorescence quantification of lipofuscin in N2 worms pretreated with control or TSG. The experiment was repeated three times for each group. ** *p* ≤ 0.01, *** *p* ≤ 0.001 by Student’s *t* test using Prism 8.0.

To extensively elucidate the positive health benefits of main constituent TSG in PMT-E, we found that 200 μM TSG could significantly prolong the mean lifespan of N2 worms by 16.48% (Figure 1A and Table S3), which suggested that TSG is the major factor involved in the longevity effect of the PMT-E extract (50 μg/mL PMT-E resulted in a 19.82% increase in the mean lifespan of N2 worms) [5]. A total of 200 μM TSG significantly increased the number of body bends of N2 worms within 30 s by 26.34% on the 8th day of adulthood (Figure 1B and Table S4), and clearly increased the frequency of pharyngeal pumps within 15 s in worms on the 4th and 8th days of adulthood by 8.95% and 15.44%, respectively (Figure 1C and Table S5). Additionally, Lipofuscin is an important characteristic of nematode senescence [13]. We found that the fluorescence intensity of the worms treated with 200 μM TSG was obviously decreased by 26.51% compared with the control group (Figure 1D,E), indicating that TSG effectively inhibited the accumulation of lipofuscin in vivo.

Taken together, these data demonstrated that supplementation with TSG not only prolonged the lifespan of worms but also improved their health status by delaying age-associated physiological decline in N2 worms.

### 3.2. TSG Protected against Oxidative Stress in C. elegans

**Figure 2:**
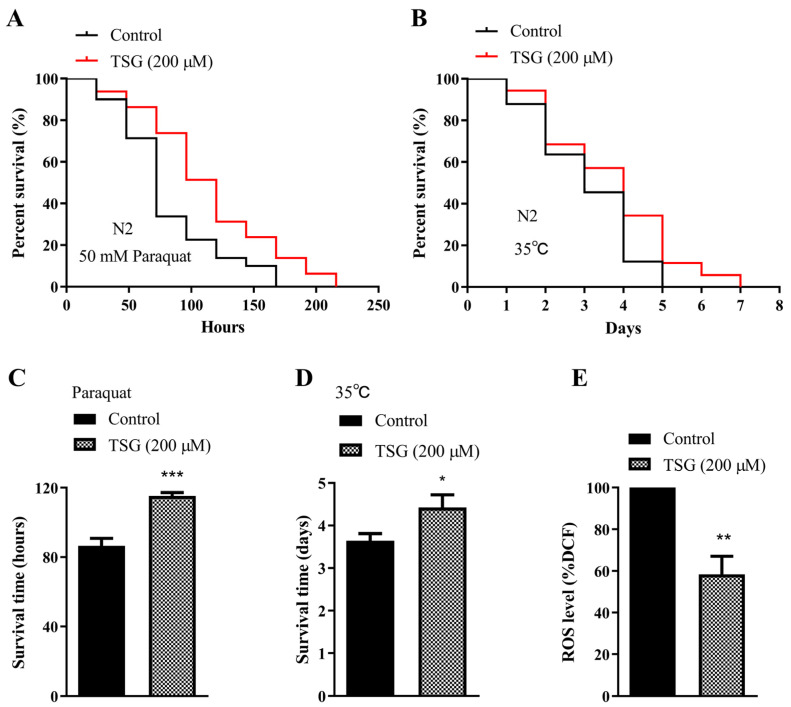
TSG enhanced stress resistance and attenuated intracellular ROS levels in wild-type worms. (**A**,**C**) TSG treatment increased the lifespan of 4-day-old adult N2 worms under 50 mM paraquat conditions. (**B**,**D**) TSG effectively promoted the thermal stress resistance of nematodes. Pretreated 4-day-old adult worms were incubated at 35 °C for 4 h, after which the survival rate was scored. In (**C**,**D**), the mean lifespan of worms exposed to oxidative and thermal stress is shown. (**E**) TSG decreased ROS accumulation at day 4 of adulthood. The intracellular ROS level was measured by the fluorescent probe H2DCF-DA. The experiment was repeated three times for each group. * *p* ≤ 0.05, ** *p* ≤ 0.01, *** *p* ≤ 0.001, as determined by Student’s *t* test.

Prolonged longevity in worms is generally associated with enhanced stress resistance. We found that 200 μM TSG treatment increased the percentage of surviving worms under 50 mM paraquat-induced oxidative stress by 33.20% (Figure 2A,C and Table S6). The average survival time of the nematodes treated with 200 μM TSG was increased by 21.41% compared with that of the control group after thermal stimulation at 35 °C for 4 h (Figure 2B,D and Table S7). Furthermore, TSG treatment obviously decreased the level of intracellular ROS by 41.57%, as measured using H2DCF-DA (Figure 2E). These findings suggested that the lifespan extension induced by TSG was associated with its antioxidative activity.

### 3.3. TSG Improved Mitochondrial Function

**Figure 3:**
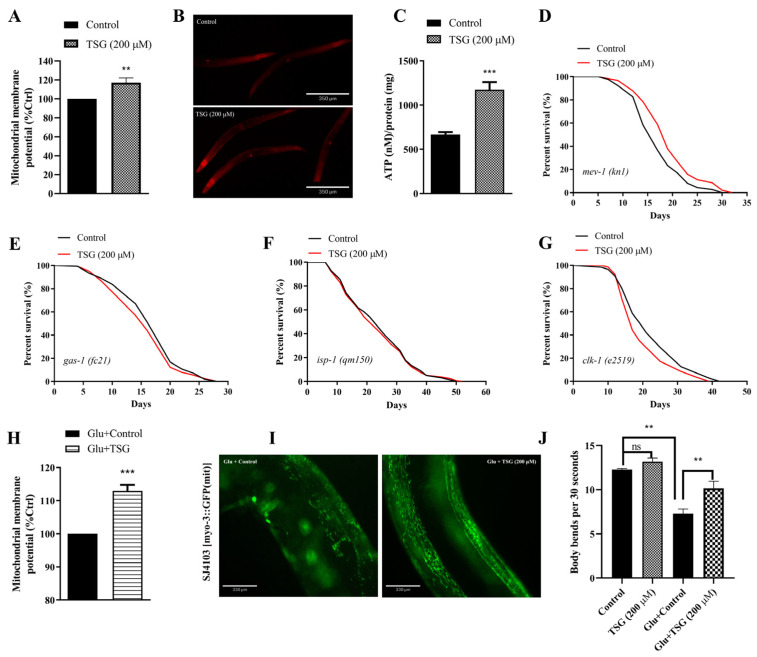
TSG improved mitochondrial function. (**A**,**B**) TSG enhanced the mitochondrial membrane potential (MMP). (**C**) TSG increased ATP levels in *C. elegans*. (**D**–**G**) TSG-induced longevity requires the regulation of mitochondrial electron transport chain (ETC) function. In (**D**–**F**), TSG decreased or did not affect the lifespan of ETC mutants such as *gas-1 *(*fc21*), *isp-1 *(*qm150*) or *clk-1 *(*e2519*). In (**G**), TSG treatment increased the lifespan of the *mev-1 *(*kn1*) mutant. (**H**) TSG increased mitochondrial membrane potential under the condition of 50 mM high glucose. (**I**) TSG protected the morphology of the mitochondrial network and reduced the fragmentation caused by high glucose. (**J**) TSG promoted motility in nematodes under high-glucose stress. The above experiments were performed at 4-day-old adulthood. Survival data (**D**–**G**) were compared using the log-rank test, and other data were analyzed by Student’s *t* test. ns, not significant; ** *p* ≤ 0.01, *** *p* ≤ 0.001.

The aging process causes mitochondrial dysfunction, which is characterized by electron leakage from the respiratory chain and excess ROS production [14]. We found that 200 μM TSG treatment increased the mitochondrial membrane potential (ΔΨ) by staining active mitochondria with rhodamine 6G fluorescent probes (Figure 3A,B). Additionally, ATP production was also increased in worms treated for 96 h with TSG (Figure 3C).

To further investigate whether increased mitochondrial activity is involved in TSG-mediated longevity, the lifespans of individuals with four mutations in mitochondrial ETC complexes, such as *gas-1*, *mev-1*, *isp-1* and *clk-1*, were measured. We observed that the mean lifespan of the *mev-1* mutant overexpressing superoxide from the cytochrome b large subunit of complex II was extended by TSG (Figure 3D). However, the ability of TSG to improve lifespan disappeared entirely in *gas-1* (homologous to human NDUFS2, encodes the core subunit of complex I), *isp-1* (encoding the iron–sulfur protein of complex III) and *clk-1* (encoding an enzyme that is necessary for the biosynthesis of coenzyme Q) mutants (Figure 3E–G), indicating that life extension by TSG was partially dependent on the activation of mitochondria.

Moreover, we investigated the effect of TSG on acute mitochondrial dysfunction under high-glucose stress. The results showed that TSG could also increase mitochondrial membrane potential under the condition of 50 mM high glucose (Figure 3H). The mitochondrial morphology was changed from reticular to bulky with a shortened length and fragmented state under a high-glucose environment (Figure 3I), but the TSG-treated group exhibited reduced fragmentation and displayed more tubular morphology in the body wall muscle cells, indicating that TSG effectively inhibited the mitochondrial dysfunction induced by high glucose. Furthermore, TSG significantly increased the body bending frequency of 4-day-old worms by 39.56% under high-sugar conditions (Figure 3J), suggesting that TSG could inhibit the decrease in exercise ability caused by a high-glucose diet and effectively delay the aging rate of nematodes. These data strongly support a pivotal role for enhanced mitochondrial health in TSG-induced longevity extension.

### 3.4. TSG Improves Mitochondrial Function by Inducing Mitophagy and Stimulating Mitochondrial Biogenesis

**Figure 4:**
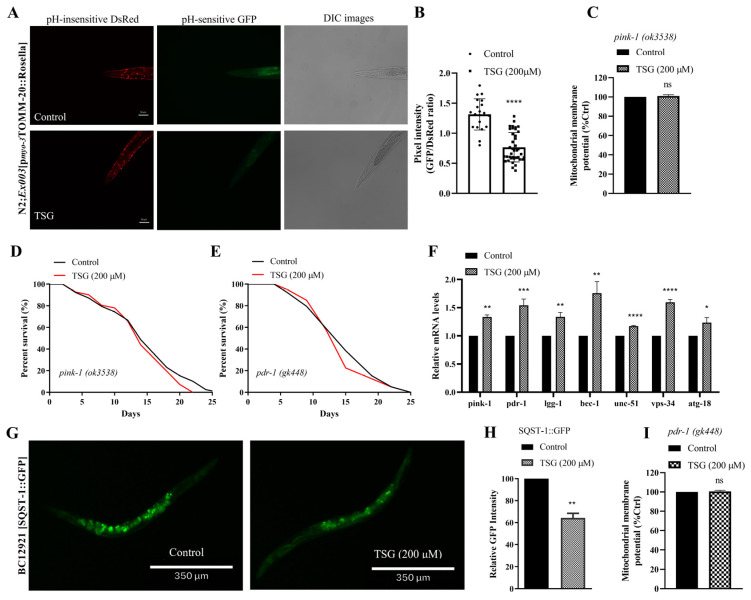
TSG promoted PINK1-PDR1-dependent mitophagy. (**A**,**B**) TSG activated mitophagy in transgenic animals expressing the mtRosella biosensor in body wall muscle cells. A reduced ratio between pH-sensitive GFP and pH-insensitive DsRed indicated an increase in mitophagy. (**C**,**I**) TSG did not increase the MMP in *pink-1 *(*ok3538*) or *pdr-1 *(*gk448*) mutants. (**D**,**E**) *pink-1* or *pdr-1* deletion eliminated TSG-induced lifespan extension. (**F**) TSG increased the expression of mitophagy-related genes (*pink-1*, *pdr-1*, *lgg-1*, *bec-1*, *unc-51*, *vps-34* and *atg-18*). (**G**,**H**) TSG decreased the SQST-1::GFP fluorescence intensity in BC12921 (SQST-1::GFP)strains. Each experiment was repeated at least three times. ns, not significant; * *p* ≤ 0.05, ** *p* ≤ 0.01, *** *p* ≤ 0.001, **** *p* ≤ 0.0001.

Given the significant ability of TSG to protect mitochondrial function, we further examined whether TSG regulates mitochondrial quality control processes, including mitophagy and mitochondrial biogenesis, which are responsible for mitochondrial homeostasis [15]. To investigate the role of mitophagy in TSG-induced health benefits, we examined animals expressing the mtRosella biosensor that combines a pH-insensitive DsRed fused to a pH-sensitive GFP variant [16]. The data showed that 200 μM TSG treatment reduced the GFP/DsRed ratio of mtRosella fluorescence, indicating the stimulation of mitophagy (Figure 4A,B). Additionally, we assessed the lifespan and mitochondrial function of worms deficient in *pink-1* and *pdr-1*, which are critical factors for activating mitophagy. TSG did not increase the survival or mitochondrial membrane potential in mutants in *pink-1 *(*ok3538*) or *pdr-1 *(*gk448*) (Figure 4C–E,I). Indeed, the PCR data of N2 worms suggested that TSG significantly induced the expression of mitophagy genes, including *unc-51*, *bec-1*, *vps-34*, *atg-18*, *pink-1*, *pdr-1* and *lgg-1* (Figure 4F). Moreover, the fluorescence intensity of SQST-1/p62, a well-known autophagic substrate for monitoring autophagic flux, was significantly reduced after TSG treatment (Figure 4G,H). These results further illustrated that the TSG-induced improvements in lifespan and mitochondrial function required PINK-1/PDR-1-regulated mitophagy.

**Figure 5:**
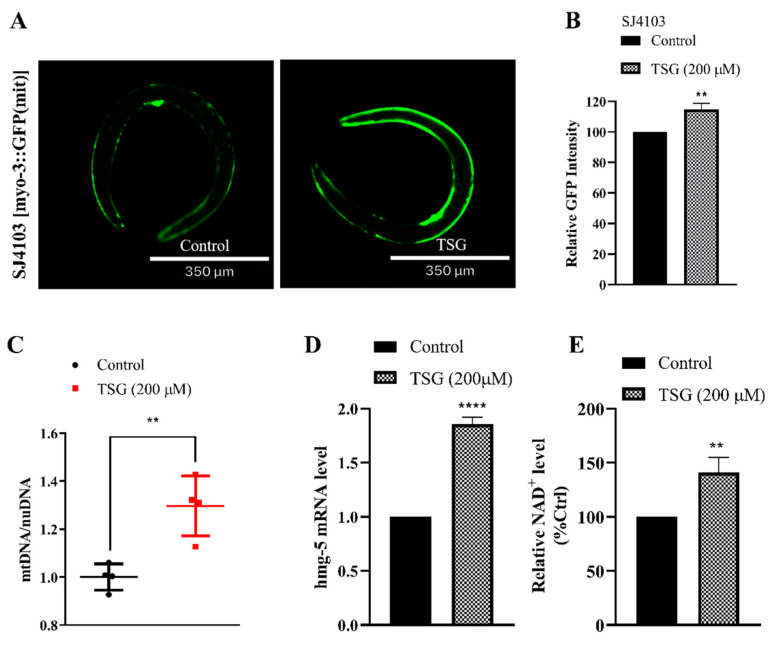
TSG stimulated mitochondrial biogenesis. (**A**,**B**) TSG increased the mitochondrial content in SJ4103 (myo-3::mtGFP) animals expressing mitochondrial-targeted GFP in the body wall muscle cells. (**C**) TSG increased the mtDNA/nDNA ratio. (**D**) TSG increased mitochondrial biogenesis gene expression, as did *hmg-5*. (**E**) TSG increased the level of NAD^+^ in *C. elegans*. ** *p* ≤ 0.01, **** *p* ≤ 0.0001 by Student’s *t* test.

In addition to mitophagy, mitochondrial biogenesis is also involved in the regulation of mitochondrial quantity. Mitochondrial biogenesis is a process that increases mitochondrial mass within cells by extensively coordinating both the mitochondrial and nuclear genomes [2,17]. There were more mitochondria in the body wall muscle of the TSG-treated myo-3::GFP(mit) strain than in that of the vehicle control strain (Figure 5A,B), which was corroborated by evaluating the mtDNA/nDNA ratio and the expression of the *hmg-5* gene functional ortholog of mammalian TFAM (Figure 5C,D). Nicotinamide adenine dinucleotide (NAD^+^) can increase gene transcription during mitochondrial biogenesis, and its decrease is also a biomarker of mitochondrial dysfunction [18]. As shown in Figure 5E, the NAD^+^ concentration of worms treated with TSG was significantly increased.

Taken together, these results suggested that TSG improved mitochondrial homeostasis by inducing mitophagy and mitochondrial biogenesis in *C. elegans*.

### 3.5. TSG Modulated DAF-16/FOXO, SKN-1/Nrf2 and SIR-2.1/SIRT1 to Activate Mitochondrial Biogenesis and Mitophagy

**Figure 6:**
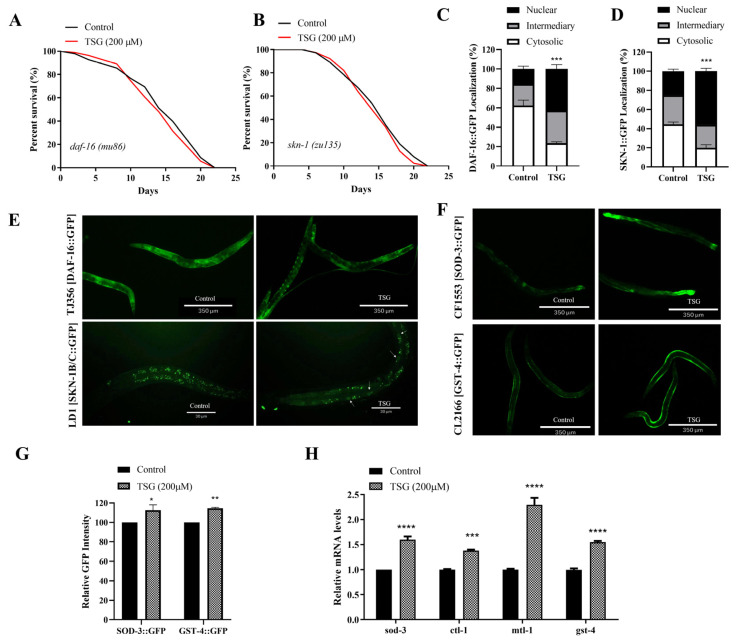
TSG regulated the antioxidant-stress signaling pathway mediated by the transcription factors DAF-16 and SKN-1. (**A**,**B**) Lifespans of *daf-16* (*mu86*) and *skn-1 *(*zu135*) mutants treated with TSG or vehicle. (**C**–**E**) TSG increased DAF-16 and SKN-1 nuclear localization. Representative images of the transgenic strains TJ356 and LD1. (**F**,**G**) Representative images and quantification of SOD-3::GFP in the CF1553 strain and GST-4::GFP in the CL2166 strain. (**H**) TSG upregulated the expression of antioxidant-related genes at day 4 of adulthood. The experiment was repeated three times for each group. In (**A**,**B**), *p* values represent comparisons with controls calculated using the log-rank test; in (**C**,**D**,**G**,**H**), * *p* ≤ 0.05, ** *p* ≤ 0.01, *** *p* ≤ 0.001, **** *p* ≤ 0.0001 according to Student’s *t* test.

Damaged or redundant mitochondria can induce oxidative stress and initiate the coordinated induction of genes involved in mitochondrial biogenesis and mitophagy mediated by insulin/IGF signals and SKN-1 [16]. We found that both the increase in protection against rotenone and the increase in the mitochondrial membrane potential induced by TSG treatment were eliminated in the *daf-16 *(*mu86*) and *skn-1 *(*zu135*) strains (Figure S1A,B,D,E), and the mRNA levels of mitochondrial-related genes were not changed in *daf-16* and *skn-1* mutants treated with TSG (Figure S1C,F), which indicating that TSG-regulated mitochondrial quality control was dependent on DAF-16 and SKN-1. In addition, SKN-1 and DAF-16 play a key role in antioxidant defense and senescence. We found that the mean lifespans of the *daf-16 *(*mu86*) and *skn-1 *(*zu135*) mutants were not significantly extended after TSG treatment compared with those of the control (Figure 6A,B). The nuclear localization of DAF-16 and SKN-1 in worms were increased from 15.46% to 42.73% and 25.12% to 56.00%, respectively, after TSG treatment (Figure 6C–E). These results were confirmed at higher expression levels of their downstream target genes, such as *sod-3* and *gst-4*, by qRT–PCR and the quantification of the GFP intensity in the CF1553 and CL2166 strains (Figure 6F–H). Taken together, these data suggested that the transcription factors DAF-16 and SKN-1 are involved in TSG-mediated lifespan extension by increasing the mitochondrial function.

**Figure 7:**
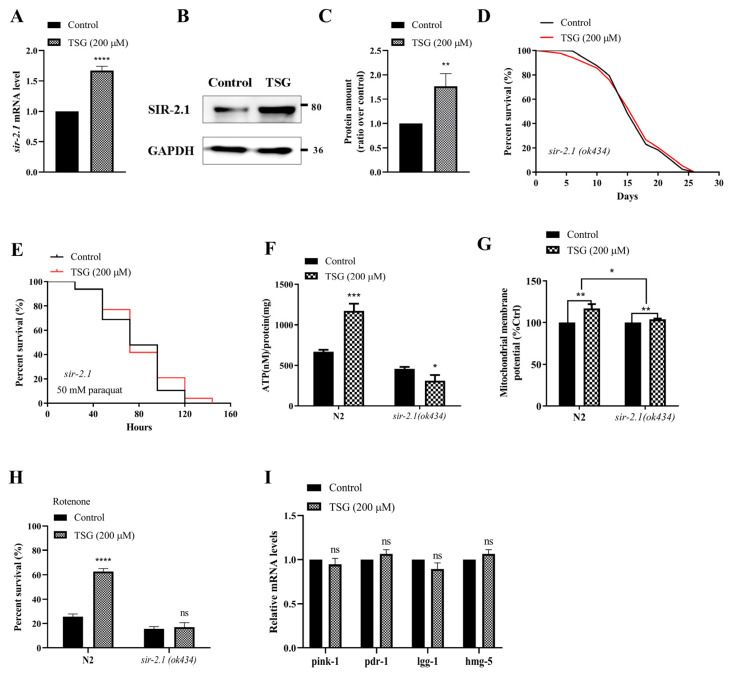
TSG modulates SIR-2.1/SIRT1 to activate mitochondrial biogenesis and mitophagy. (**A**) TSG increased the *sir-2.1* mRNA level at day 4 of adulthood. (**B**,**C**) TSG increased the protein abundance of SIR-2.1. Representative Western blot and the quantification of bands in the gel. (**D**,**E**) TSG did not affect the lifespan of the *sir-2.1 *(*ok434*) mutant under normal or 50 mM paraquat conditions. (**F**) SIR-2.1 is required for the TSG-induced increase in ATP levels in *C. elegans*. (**G**) TSG increased the mitochondrial membrane potential in wild-type animals, but this effect was attenuated in the *sir-2.1 *(*ok434*) mutant. (**H**) TSG increased the survival percentage of nematodes in the presence of 50 μM rotenone in a manner dependent on *sir-2.1*. (**I**) The induction of mitophagy and mitochondrial biogenesis gene expression by TSG was also *sir-2.1* dependent. ns, not significant; * *p* ≤ 0.05, ** *p* ≤ 0.01, *** *p* ≤ 0.001, **** *p* ≤ 0.0001.

TSG appears to delay symptoms in aging mice by affecting the SIRT1, which has NAD^+^-dependent deacetylase activity to catalyze the deacetylation of p53, FOXO and PGC-1α proteins [19]. Considering that TSG increased the NAD^+^ content in N2 worms according to the above results, we speculate that the sirtuin pathway may also be involved in the improvement of mitochondrial function and lifespan extension by TSG in *C. elegans*. Our results showed that the gene transcription and protein expression levels of the sirtuin homologous gene *sir-2.1* were significantly increased in TSG-treated N2 worms (Figure 7A–C). Furthermore, TSG treatment could not improve the survival rate of the *sir-2.1* mutant under either normal or paraquat-induced oxidative stress conditions (Figure 7D,E). These results suggested that sirtuin activation is required for TSG-mediated lifespan.

We finally investigated the role of the sirtuin signaling pathway in TSG-mediated mitochondrial function. As expected, a lower or no effect on ΔΨ and ATP was observed in the TSG-treated *sir-2.1* mutants (Figure 7F,G). The rotenone-induced survival rate of *sir-2.1 *(*ok434*) mutants was not changed after TSG treatment (Figure 7H). Moreover, the qRT–PCR results showed that there was no significant difference in the mRNA expression levels of *pink-1*, *pdr-1*, *lgg-1* and *hmg-5* in *sir-2.1 *(*ok434*) mutants after TSG administration (Figure 7I), suggesting that *sir-2.1* is necessary for the mitophagy and mitochondrial biogenesis induced by TSG.

Collectively, these data revealed that TSG coordinated mitophagy and mitochondrial biogenesis to improve mitochondrial function and highlighted the critical role of DAF-6/SKN-1/SIR-2.1 in the TSG-mediated regulation of mitochondrial quality control.

### 3.6. TSG Protected against Aβ- or Tau-Induced Toxicity

**Figure 8:**
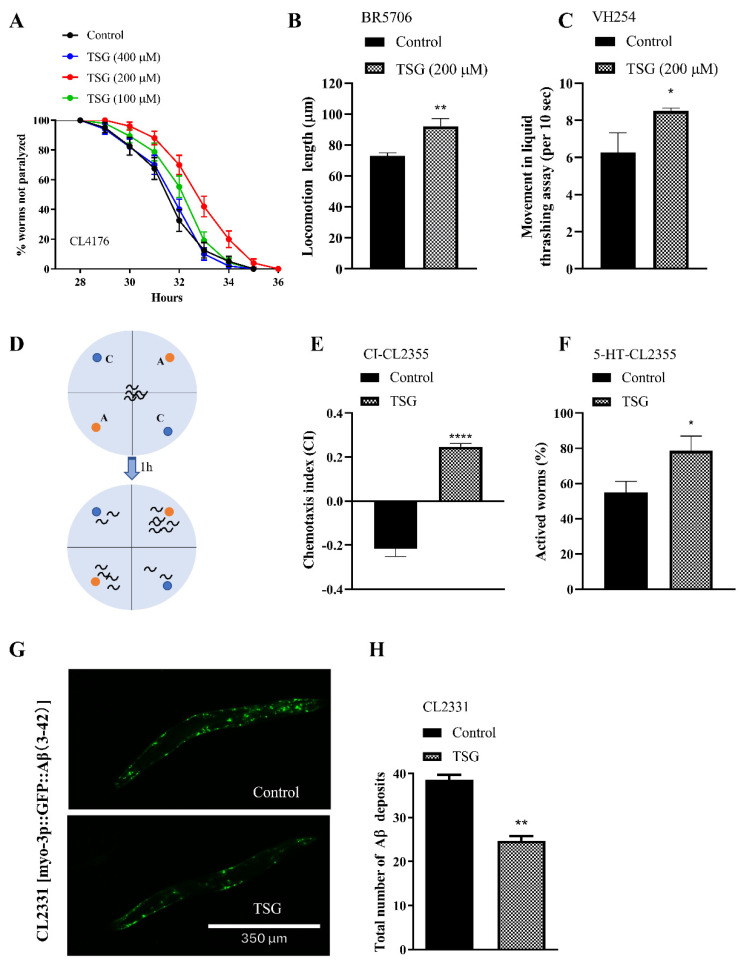
TSG improved motor impairment and neuronal damage caused by the Aβ and Tau proteins in AD transgenic worms. (**A**) TSG alleviated Aβ-induced paralysis in CL4176 worms. This strain could induce the expression of human Aβ_1-42_ by temperature upregulation. (**B**) TSG increased the locomotion length of the tau transgenic nematode BR5706 within 10 s. (**C**) TSG increased the thrashing frequency of the tau transgenic worm VH254 within 10 s at day 4 of adulthood. (**D**,**E**) TSG attenuated the chemical attractant searching deficit in CL2355 worms. Chemical index (CI) = number of animals at attractant–number of animals at water location/total number of animals. A: attractant, C: control. (**F**) TSG enhanced the survival of 5-HT-treated worms. (**G**,**H**) TSG reduced Aβ aggregation in the CL2331 strain expressing Aβ_3-42_ coupled with GFP. Each experiment was repeated at least three times. * *p* ≤ 0.05, ** *p* ≤ 0.01, **** *p* ≤ 0.0001.

Compounds with prolongevity and anti-oxidative effects have the potential to protect against aging-related diseases, such as Alzheimer’s disease (AD) [20]. We observed that TSG significantly delayed paralysis by approximately 35.57% in CL4176 worms expressing human Aβ in body wall muscle (Figure 8A and Table S8). Furthermore, treatment with TSG improved the locomotion length and thrash rates of tau transgenic BR5706 and VH254 *C. elegans* by 26.41% and 39.06%, respectively (Figure 8B,C), suggesting that TSG could obviously suppress the toxicity induced by Aβ and tau in muscle cells.

To demonstrate the inhibitory effect of TSG on Aβ toxicity to neurons, we evaluated memory retention in terms of chemotaxis behavior and 5-HT sensitivity in the CL2355 strain, which induces Aβ expression in pan-neurons. As shown in Figure 8D,E, TSG obviously increased the chemical index (CI), indicating that TSG attenuated the chemical attractant searching deficit in CL2355 worms. TSG also markedly reversed the defective response to 5-HT by enhancing survival in the 5-HT sensitivity assay (Figure 8F). Overall, TSG effectively inhibited Aβ-induced neuronal injury, improving memory deficits.

Additionally, TSG treatment significantly decreased the number of Aβ deposits of CL2331 strain by 35.84% (Figure 8G,H), which expresses GFP-coupled Aβ in body wall muscle cells, indicating that TSG alleviated Aβ-induced paralysis and neurotoxicity by reducing the accumulation of Aβ in *C. elegans*.

### 3.7. TSG Inhibited Aβ-Induced Toxicity through Antioxidant Activity and Improving Mitochondrial Function

**Figure 9:**
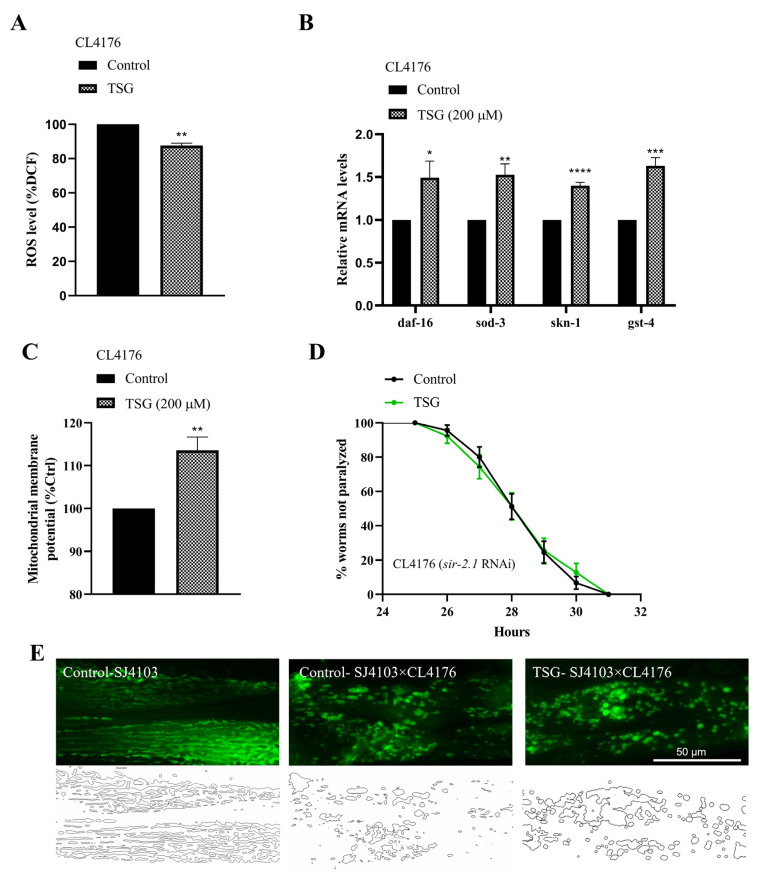
TSG-mediated antioxidation and mitochondrial function protection against Aβ-induced toxicity. (**A**) TSG reduced the ROS level in CL4176. (**B**) TSG increased the expression of antioxidant genes (*daf-16*, *skn-1*, *sod-3* and *gst-4*). (**C**) TSG enhanced the mitochondrial membrane potential in the CL4176 strain. (**D**) RNAi of *sir-2.1* abolished the delayed paralysis. (**E**) TSG reversed the morphology of the mitochondrial network manifested as inhibited fragmentation in the hybrid strain, which was generated by crossing CL4176 with the SJ4103 strain. The above experiments were performed at the 31st hour after the temperature rise. Each experiment was repeated at least three times. * *p* ≤ 0.05, ** *p* ≤ 0.01, *** *p* ≤ 0.001, **** *p* ≤ 0.0001.

Oxidative stress is one of the initiating factors involved in the pathogenetic cascade of AD. We measured the ROS levels in CL4176 worms with the H2DCF-DA probe and found that compared with the control, TSG reduced the ROS level by 12.35% (Figure 9A). In contrast, the mRNA expression levels of *daf-16* and *skn-1* and their target genes were markedly upregulated in the TSG treatment group (Figure 9B), emphasizing that an antioxidant-stress signaling pathway regulated by the transcription factors DAF-16 and SKN-1 was involved in the protective effect of TSG against Aβ toxicity.

The abnormal distribution and dysfunction of mitochondria induced by Aβ are important causes of cognitive impairment [21]. Our data showed that the TSG treatment not only significantly increased the membrane potential (Figure 9C) but also effectively restored the morphology of the mitochondrial network manifested as inhibited fragmentation in the hybrid strain, which was generated from the cross of CL4176 with the SJ4103 strain (Figure 9E). We also revealed that the delayed paralysis caused by TSG can be abolished with *sir-2.1* RNAi (Figure 9D). Collectively, our data suggested that TSG improved mitochondrial function and alleviated the AD paralysis phenotype in a *sir-2.1*-dependent manner.

## 4. Discussion

Accumulating evidence has suggested a causative link between mitochondrial dysfunction and major phenotypes associated with aging [14,22]. We explored the effect of TSG on mitochondrial quality control (MQC), a conserved system at the molecular and organelle levels, as an indispensable defense mechanism against mitochondrial dysfunction [23]. Mitophagy is used to eliminate abnormal mitochondrial proteins and part of the mitochondrial network to prevent the accumulation of damaged or excess mitochondria, while the protein and lipid components of mitochondria can be updated via mitochondrial biogenesis, which enhances the capacity for mitochondrial oxidative phosphorylation and ATP synthesis [24]. Overall, the coordination of mitophagy and mitochondrial biogenesis is an intervention measure for maintaining energy metabolism homeostasis. Our results suggested that TSG improved mitochondrial function by activating both mitophagy and mitochondrial biogenesis. In fact, resveratrol, which has a structure similar to that of TSG, has been reported to effectively ameliorate mitochondrial damage caused by myocardial ischemia/reperfusion injury via this balancing mechanism [25]. Moreover, tomatidine also relies on the above mechanisms to improve mitochondrial function and delay senescence in *C. elegans* and human cells [26]. Therefore, drug design and screening to improve mitochondrial function through mitochondrial quality regulatory mechanisms may be a potential strategy for delaying aging and treating senile diseases.

Our findings resonate with previous reports that the inhibition of insulin/IGF-1 signaling and the activation of Nrf2 or sirtuins are directly or indirectly linked to mitochondrial biogenesis and mitophagy. The impairment of mitophagy functions, such as with PINK-1 and PDR-1 RNAi, could abrogate the elevated autophagy in *daf-2 *(*e1370*) mutants and markedly shorten their lifespan under these conditions [16]. Preserved muscle function in aging *daf-2 *(*e1370*) mutants is also associated with increased muscle mitochondrial mass, preserved mitochondrial morphology, and increased levels of intracellular ATP [27]. Furthermore, SKN-1 transcriptional activity is essential for the response to mitochondrial dysfunction and drives the expression of several mitochondrial biogenesis genes [16]. Additionally, emerging evidence suggests that sirtuins have dual functions in regulating mitochondrial biogenesis and mitophagy in mammals. For example, the activation of the SIRT1-PGC-1α axis is a familiar pathway for regulating mitochondrial biogenesis [28]. The SIRT1-FOXO3-PINK1-Parkin signaling pathway is capable of participating in the regulation of mitophagy and clearing damaged mitochondria to protect renal function [29].

Our findings also revealed that α-synuclein and polyQ aggregation are significantly reduced in TSG-treated Parkinson’s disease and Huntington’s disease models, and TSG had a neuroprotective effect on dopaminergic neuronal damage induced by 6-OHDA (Figure S2). Therefore, the protective effects of TSG on oxidative stress and mitochondrial function, as well as its protection against toxicity caused by Aβ, tau, α-Syn and polyQ misfolded proteins, suggested that targeting the aging process could prevent a range of age-related neurodegenerative diseases. It was reported that the activation of DAF-16 alleviated Aβ_1-42_-induced paralysis and induced autophagy to degrade protein aggregates [30], while SKN-1 regulated the endoplasmic reticulum unfolded protein response (UPR^ER^) and participated in proteasome subunit synthesis, maintaining protein homeostasis by reducing misfolding and degradation pathways [31]. Notably, TSG-dependent *sir-2.1*-mediated protection of mitochondrial function not only prolonged the lifespan of wild-type worms but also inhibited the Aβ toxicity of AD nematodes, which is consistent with a study showing that the knockout of the SIRT1 protein induces neurodegenerative diseases in aging mice, while the overexpression of the SIRT1 protein effectively reduced the formation of amyloid plaques and improved the behavioral phenotype [32]. Hence, these observations emphasized that antioxidants and improved mitochondrial function played important roles in the TSG-mediated inhibition of neurodegeneration.

In this study, the aging research mainly used the liquid culture method, which can save drugs and reduce the abnormal death of nematode (e.g., worms climbing to the side wall of the Petri dish, resulting in dehydration) compared with solid culture [33]. Since the liquid medium contains FUDR almost all the time and recent studies have reported that FUDR affects the lifespan of worms through reproductive signaling pathways [34], in order to avoid the interference of FUDR on the experimental results, we further examined the effects of TSG on oviposition and offspring hatching. There was no significant difference in the total number of offspring between the TSG and the control group (Figure S3), which indicates that TSG did not interfere with reproduction or the development of progeny. In addition, permeability may also be involved in the life-extending effect of TSG [35,36]. Therefore, the experiments on the resistance to osmotic stress, intestinal permeability and the regulation of the osmotic-pressure signaling pathway deserve further study.

In summary, the results obtained in our study indicate that improving mitochondrial function via supplementation with TSG is a promising approach for extending longevity and alleviating the proteotoxicity of misfolded proteins associated with aging. These findings provide a foundation for the therapeutic application of TSG in aging and aging-related diseases.

## Data Availability

Data are available within the article or Supplementary Materials.

## Supplementary Materials

The following supporting information can be downloaded at: https://www.mdpi.com/article/10.3390/antiox13091086/s1.

## Abbreviations

TSG	2,3,5,4′-Tetrahydroxystilbene-2-*O*-*β*-D-glucoside;
*C. elegans*	*Caenorhabditis elegans*;
ETC	electron transfer chain;
MQC	mitochondrial quality control;
UPR^mt^	mitochondrial unfolded protein response;
PMT-E	ethanol fraction of Polygonum multiflorum Thunb;
Aβ	β-amyloid;
NGM	nematode growth media;
CGC	Caenorhabditis Genetics Center;
CI	chemotaxis index;
ΔΨ	mitochondrial membrane potential;
UPR^ER^	reticulum unfolded protein response;
AD	Alzheimer’s disease.
