# Supplementary material for: TSG Extends the Longevity of Caenorhabditis elegans by Targeting the DAF-16/SKN-1/SIR-2.1-Mediated Mitochondrial Quality Control Process
Source: Antioxidants (Basel). 2024 Sep 4;13(9):1086. doi: 10.3390/antiox13091086 (PMC11428426; doi:10.3390/antiox13091086)
Supplement: Supplementary file 1 [file antioxidants-13-01086-s001.zip › antioxidants-3111734-supplementary.pdf]

Supplementary Materials

TSG extends the longevity of *Caenorhabditis elegans* by targeting the DAF-16/SKN-1/SIR-2.1-mediated mitochondrial quality control process

Table S1. List of the *C. elegans* strains.

Table S2. The primers used for qPCR.

Table S3. Summary of *C. elegans* lifespan experiments.

Table S4. TSG increased the body bending rate of N2 wild-type *C. elegans.*

Table S5. TSG increased the pharyngeal pumping rate of N2 wild-type *C. elegans*.

Table S6. Effect of TSG on oxidative stress resistance of different strain *C. elegans*.

Table S7. Effect of TSG on heat stress resistance of N2 wild-type worms.

Table S8. TSG ameliorated Aβ-induced paralysis of transgenic *C. elegans* strain CL4176.

Figure S1. Effects of TSG on mitochondrial in *daf-16 (mu86)* and *skn-1 (zu135)* mutants.

Figure S2. TSG decreased α-synuclein and polyQ-mediated neurotoxicity in PD and HD models.

Figure S3. TSG did not impact production in *C. elegans*.

Table S1 List of the *C. elegans* strains.

N2	Wild-type Bristol
CF1038	*daf-16 (mu86)*I
EU31	*skn-1 (zu135)*IV
CW152	*gas-1 (fc21)*X
TK22	*mev-1 (kn1)* III
MQ887	*isp-1 (qm150)*IV
CB4876	*clk-1 (e2519)* III
RB2547	*pink-1 (ok3538)*II
VC1024	*pdr-1 (gk448)* III
TJ356	(zIs356) [*daf-16p*::*daf-16a/b*::GFP + *rol-6(su1006)*]
LD1	(ldIs7) [*skn-1b/c*::GFP + *rol-6(su1006)*]
CF1553	(muIs84) [(pAD76) *sod-3p*::GFP + *rol-6(su1006)*]
CL2166	(dvIs19) [(pAF15) *gst-4p*::GFP::NLS]
SJ4103	(zcIs14) [*myo-3*::GFP(mit)]
BC12921	(sIs10729) [rCes T12G3.1::GFP + pCeh361]
CL4176	(dvIs27) [*myo-3p*::A-Beta (1-42)::let-851 3'UTR) + rol-6(su1006)]
BR5706	(byIs193) [rab-3p::F3(delta)K280 + myo-2p::mCherry]
VH254	(hdEx81) [F25B3.3::tau352(PHP) + pha-1(+)]
CL2355	(dvIs50) [pCL45 (snb-1::Abeta 1-42::3' UTR(long) + mtl-2::GFP]
CL2331	(dvIs37) [*myo-3p*::GFP::A-Beta (3-42) + *rol-6(su1006)*]
BZ555	(egIs1) [*dat-1p*::GFP]
OW13	(pkIs2386) [*unc-54p*::alpha-synnuclein::YFP + *unc-119*(+)]
AM140	(rmIs132) [*unc-54p*::Q35::YFP]
IR1631	N2;*Ex003*[$p_{myo\text{-}3}$TOMM-20::Rosella]

Table S2. The primers used for qPCR.

actin-1	forward 5' CCAGAAGAGCACCCAGTC 3'	reverse 5' TGATGTCACGGACGATTT 3'
daf-16	forward 5' TCCTCATTCACTCCCGATTC 3'	reverse 5' CCGGTGTATTCATGAACGTG 3'
sod-3	forward 5' GGATGGTGGAGAACCTTCAA 3'	reverse 5' AAGGATCCTGGTTTGCACAG 3'
ctl-1	forward 5' CGGATACCGTACTCGTGATGAT 3'	reverse 5' CCAAACAGCCACCCAAATCA 3'
mtl-1	forward 5' TGCAAGTGCGGAGACAAATG 3'	reverse 5' GTTCCCTGGTGTTGATGGGT 3'
skn-1	forward 5' CACGCCGTCAGCGAAGTA 3'	reverse 5' ATGCTCGGTGAGTATTGG 3'
gst-4	forward 5' ACCAGCCCGTGATGATTTCT 3'	reverse 5' ATCCTTTCTTGTTGCCACGT 3'
sir-2.1	forward 5' GACTTGCAACGAGCCGATTC 3'	reverse 5' GCGTTTTTCAATGGGATTTGGTG 3'
hmg-5	forward 5' CAAGTAGACGCCACGCAAAG 3'	reverse 5' GCCTTCCATTGAGCAACAGC 3'
pink-1	forward 5' GGCCAACCTAGCTCCACATT 3'	reverse 5' TGTGGCAGGTGTGTCAAGTT 3'
pdr-1	forward 5' AGCCACCGAGCGATTGATTGC 3'	reverse 5' GTGGCATTTTGGGCATCTTCTTG 3'
unc-51	forward 5' CGCCGGTGGTTCAGCGGATT 3'	reverse 5' TATCCTGGGTGTCGGCGGGG 3'
lgg-1	forward 5' GCCGAAGGAGACAAGATCCG 3'	reverse 5' GGTCCTGGTAGAGTTGTCCC 3'
atg-18	forward 5' AGCAAGCATATCCGTTTAGACC 3'	reverse 5' TTTTGAGGCTGCTTGGGACA 3'
bec-1	forward 5' AAAGCTGCGTGTGCTCAATG 3'	reverse 5' TTGGGGAAAAGGCAGAATTCCA 3'
vps-34	forward 5'TCATCCGGCAGTTCGTGCATATTC 3'	reverse 5' TCAGGCAGTTGTTGACCTTGTTCG 3'
atfs-1	forward 5' AAAAACGTGACGCTGGAAGC 3'	reverse 5' TGGTGGCATTCCGTACTCAT 3'

Table S3. Summary of *C. elegans* lifespan experiments.

Strain	Treatment (μM)	Mean lifespan ± SEM (Days)	Number of worms	Change in lifespan [a]	*P* value
N2	Control	17.66 ± 0.37	144		
	TSG-400	18.15 ± 0.34	162	2.77%	0.257
	TSG-200	20.57 ± 0.41	165	16.48%	0.006
	TSG-100	19.43 ± 0.06	157	10.02%	0.203
daf-16 (mu86)	Control	13.77 ± 0.50	350		
	TSG	14.25 ± 0.09	340	3.49%	0.448
skn-1 (zu135)	Control	15.39 ± 0.20	111		
	TSG	14.89 ± 0.44	114	-3.25%	0.366
gas-1 (fc21)	Control	16.64 ± 0.20	322		
	TSG	15.74 ± 0.21	300	-5.41%	0.088
mev-1 (kn1)	Control	16.82 ± 0.12	315		
	TSG	19.48 ± 0.41	395	15.81%	0.003
isp-1 (qm150)	Control	23.76 ± 0.12	349		
	TSG	23.02 ± 0.15	302	-3.11%	0.058
clk-1 (e2519)	Control	23.22 ± 0.37	323		
	TSG	20.55 ± 0.24	330	-11.50%	0.004
pink-1 (ok3538)	Control	14.97 ± 0.59	120		
	TSG	14.24 ± 0.24	123	-4.88%	0.3165
pdr-1 (gk448)	Control	15.01 ± 0.29	115		
	TSG	15.09 ± 0.61	119	0.53%	0.912
sir-2.1 (ok434)	Control	16.88 ± 0.10	303		
	TSG	17.17 ± 0.49	315	1.72%	0.595

Data were compared using log-rank test and represented as mean ± SEM.

Control: worms treated with DMSO.

TSG: worms treated with 200 μM TSG.

[a] Percentage is relative to the control.

Table S4. TSG increased the body bending rate of N2 wild-type *C. elegans*.

Treatment	Body bending rate/30sec (mean ± SD)			
	D4	Change	D8	Change
Control	12.29 ± 0.08		7.86 ± 0.01	
TSG	13.17 ± 0.29	7.16 ns	9.93 ± 0.03	26.34% ***

Data were compared using Students' *t*-test. ns, not significant; *** $P \leq 0.001$.

Table S5. TSG increased the pharyngeal pumping rate of N2 wild-type *C. elegans*.

Treatment	Pharyngeal pumping rate /15sec (mean ± SD)			
	D4	Change	D8	Change
Control	48.69 ± 0.48		43.33 ± 0.70	
TSG	53.05 ± 0.61	8.95% **	50.02 ± 0.18	15.44% ***

Data were compared using Students' *t*-test. ** $P \leq 0.01$; *** $P \leq 0.001$.

Table S6. Effect of TSG on oxidative stress resistance of different strain *C. elegans*.

Strain	Treatment (μM)	Mean lifespan ± SEM (Hours)	Number of worms	Percentage change	*p* value
N2	Control	86.56 ± 2.49	204		
	TSG	115.30 ± 1.11	200	33.20%***	0.0005
sir-2.1 (ok434)	Control	76.73 ± 2.95	143		
	TSG	75.63 ± 3.03	145	-1.43% ns	0.808

Data were compared using log-rank test. *** $P \leq 0.001$; ns, not significant.

Table S7. Effect of TSG on heat stress resistance of N2 wild-type worms.

Strain	Treatment (μM)	Mean lifespan ± SEM (Days)	Number of worms	Percentage change	*p* value
N2	Control	3.64 ± 0.10	106		
	TSG	4.42 ± 0.17	108	21.41%*	0.0169

Data were compared using log-rank test. * $P \leq 0.05$.

Table S8. TSG ameliorated Aβ-induced paralysis of transgenic *C. elegans* strain CL4176.

Strain	Treatment (μM)	PT_{50}	Number of worms	Percentage change	*p* value
CL4176	Control	3.43 ± 0.07	126		
	TSG-400	3.68 ± 0.03	120	7.29% [ns]	0.081
	TSG-200	4.65 ± 0.05	135	35.575*	0.0219
	TSG-100	4.07 ± 0.07	119	18.66%**	0.0049

Data were compared using Students' *t*-test. * $P \le 0.05$, ** $P \le 0.01$; ns, not significant.

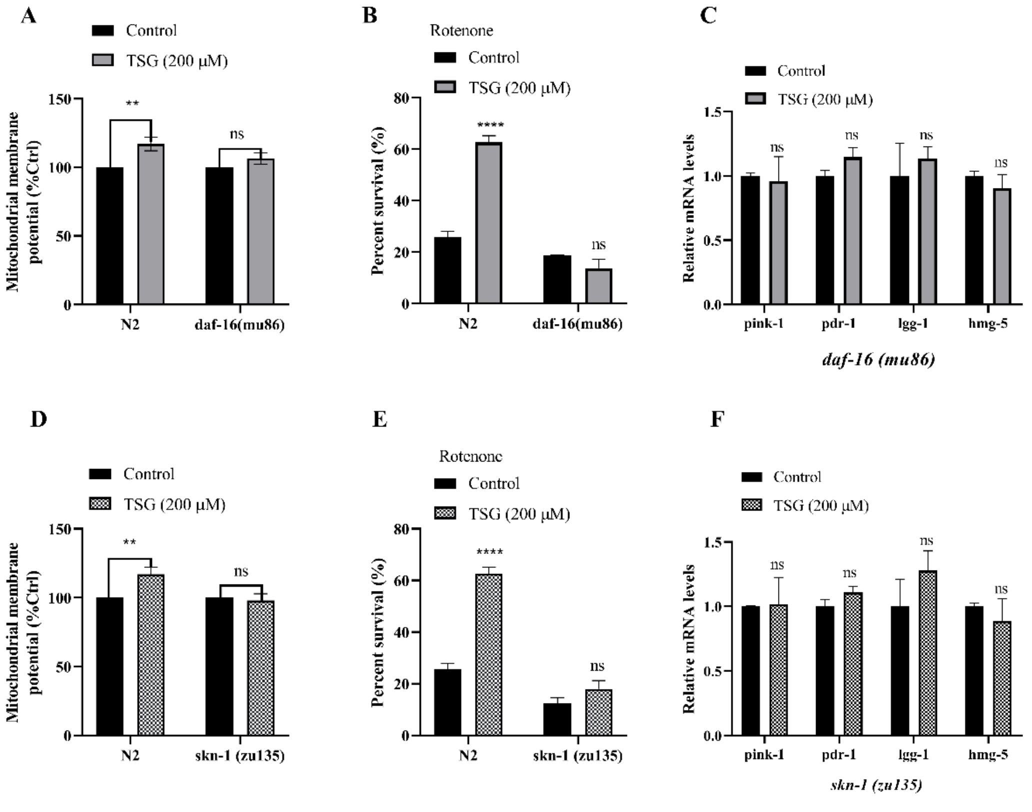

Fig. S1. Effects of TSG on mitochondrial in *daf-16 (mu86)* and *skn-1 (zu135)* mutants. (**A, D**). TSG did not increase the mitochondrial membrane potential in *daf-16 (mu86)* or *skn-1 (zu135)* mutants. **(B, E)** DAF-16 and SKN-1 were required for TSG treatment to improve the survival rate of nematodes in 50 μM rotenone. **(C, F)** TSG had no effect on the transcription of genes related to mitophagy and mitochondrial biogenesis in *daf-16 (mu86)* and *skn-1 (zu135)* mutants. Each experiment was repeated at least three times. ** $P \le 0.01$, **** $P \le 0.0001$, ns, not significant.

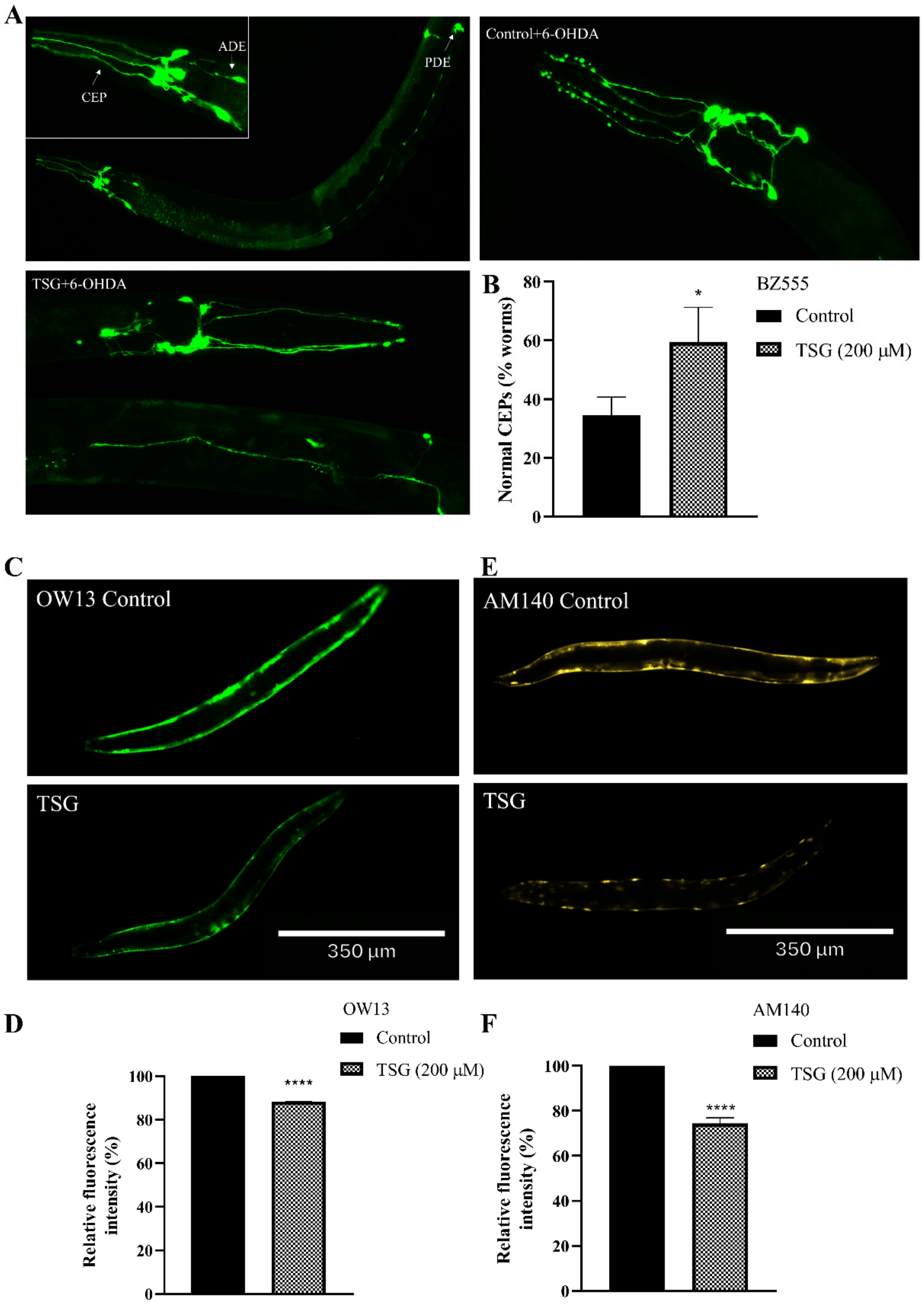

Fig. S2. TSG decreased α-synuclein and polyQ-mediated neurotoxicity in PD and HD models. (A, B) TSG alleviates 6-OHDA-induced dopaminergic neuronal degeneration in BZ555 (P_{dat}::GFP) worms. Synchronized L3 BZ555 worms were exposed to 50 mM 6-OHDA and 10 mM ascorbic acid for 1 h. The worms were subsequently washed with M9 buffer after induction and treated with DMSO or TSG for 4 days on NGM plates containing OP50. The damage was examined according to whether CEP and ADE neurons were broken or notched in the head regions. Arrows indicate CEP, ADE and PDE dopamine

neurons. **(C, D)** TSG treatment reduced the accumulation of α-synuclein in the OW13 strain. **(E, F)** TSG inhibited the aggregation of polyQ in AM140 worms. * $P \leq 0.05$, **** $P \leq 0.0001$.

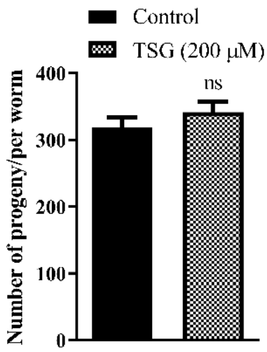

Fig. S3. TSG did not impact production in *C. elegans*. The effect of TSG on fertility was assessed by examining the total progeny. Briefly, ten synchronized N2 L4 worms were individually picked onto NGM plates supplemented with or without 200 μM TSG and allowed to lay eggs for 24 h. Adult animals were then transferred to fresh plates every day until the end of the reproductive period. Progeny worms were counted when they reached the L2 or L3 stage; ns, not significant.
